# Genomic characterisation of *Arachis porphyrocalyx* (Valls & C.E. Simpson, 2005) (Leguminosae): multiple origin of *Arachis* species with x = 9

**DOI:** 10.3897/CompCytogen.v11i1.10339

**Published:** 2017-01-09

**Authors:** Silvestri María Celeste, Alejandra Marcela Ortiz, Germán Ariel Robledo, José Francisco Montenegro Valls, Graciela Inés Lavia

**Affiliations:** 1 Instituto de Botánica del Nordeste (CONICET-UNNE, Fac. Cs. Agrarias), Sargento Cabral 2131, C.C. 209, 3400 Corrientes, Argentina; 2 Facultad de Ciencias Exactas y Naturales y Agrimensura, UNNE, Av. Libertad 5460, 3400 Corrientes, Argentina; 3 Embrapa Recursos Genéticos e Biotecnologia, Brasília, DF, Brasil

**Keywords:** Arachis, chromosomes, chromosome evolution, genetic resources

## Abstract

The genus *Arachis* Linnaeus, 1753 comprises four species with *x* = 9, three belong to the section Arachis: *Arachis praecox* (Krapov. W.C. Greg. & Valls, 1994), *Arachis palustris* (Krapov. W.C. Greg. & Valls, 1994) and *Arachis decora* (Krapov. W.C. Greg. & Valls, 1994) and only one belongs to the section Erectoides: *Arachis porphyrocalyx* (Valls & C.E. Simpson, 2005). Recently, the *x* = 9 species of section Arachis have been assigned to G genome, the latest described so far. The genomic relationship of *Arachis porphyrocalyx* with these species is controversial. In the present work, we carried out a karyotypic characterisation of *Arachis porphyrocalyx* to evaluate its genomic structure and analyse the origin of all *x* = 9 *Arachis* species. *Arachis porphyrocalyx* showed a karyotype formula of 14m+4st, one pair of A chromosomes, satellited chromosomes type 8, one pair of 45S rDNA sites in the SAT chromosomes, one pair of 5S rDNA sites and pericentromeric C-DAPI+ bands in all chromosomes. Karyotype structure indicates that *Arachis porphyrocalyx* does not share the same genome type with the other three *x* = 9 species and neither with the remaining Erectoides species. Taking into account the geographic distribution, morphological and cytogenetic features, the origin of species with *x* = 9 of the genus *Arachis* cannot be unique; instead, they originated at least twice in the evolutionary history of the genus.

## Introduction

The genus *Arachis* Linnaeus, 1753 (Leguminosae) is native to South America and comprises 82 nominal species. These species were assembled into nine sections according to morphology, geographic distribution and cross compatibility (Krapovickas and Gregory 1994, Valls and Simpson 2005, Valls et al. 2013, Santana and Valls 2015). Most of species are diploid with x = 10 (2n = 20); a few (4 species) are diploid with x = 9 (2n = 18) and the rest (5 species) are tetraploid with x =10. Three of the diploid x = 9 species belong to the section Arachis: *Arachis praecox* (Krapov. W.C. Greg. & Valls, 1994) *Arachis palustris* (Krapov. W.C. Greg. & Valls, 1994) and *Arachis decora* (Krapov. W.C. Greg. & Valls, 1994) and one belongs to the section Erectoides: *Arachis porphyrocalyx* (Valls & C.E. Simpson, 2005).

Recently, a karyotype analysis of the three x = 9 species of the section Arachis revealed that they share a common karyotype structure (Silvestri et al. 2015). This is characterised by having all metacentric chromosomes except for one submetacentric pair; the lack of the small A chromosome pair; the presence of pericentromeric C-DAPI+ bands of the same brightness, position and size in all or almost all chromosome pairs; one pair of 45S rDNA sites on the unique pair of chromosomes with secondary constriction (SAT chromosomes) and one pair of 5S rDNA sites in the chromosome pair 6. This karyotype structure differs from those that characterise the other genomes of section Arachis (A, B, D, F and K genomes), whereby the three x = 9 species of the section Arachis have been assigned to a new genome, designated by the letter G (Silvestri et al. 2015).

**Figure 1.**
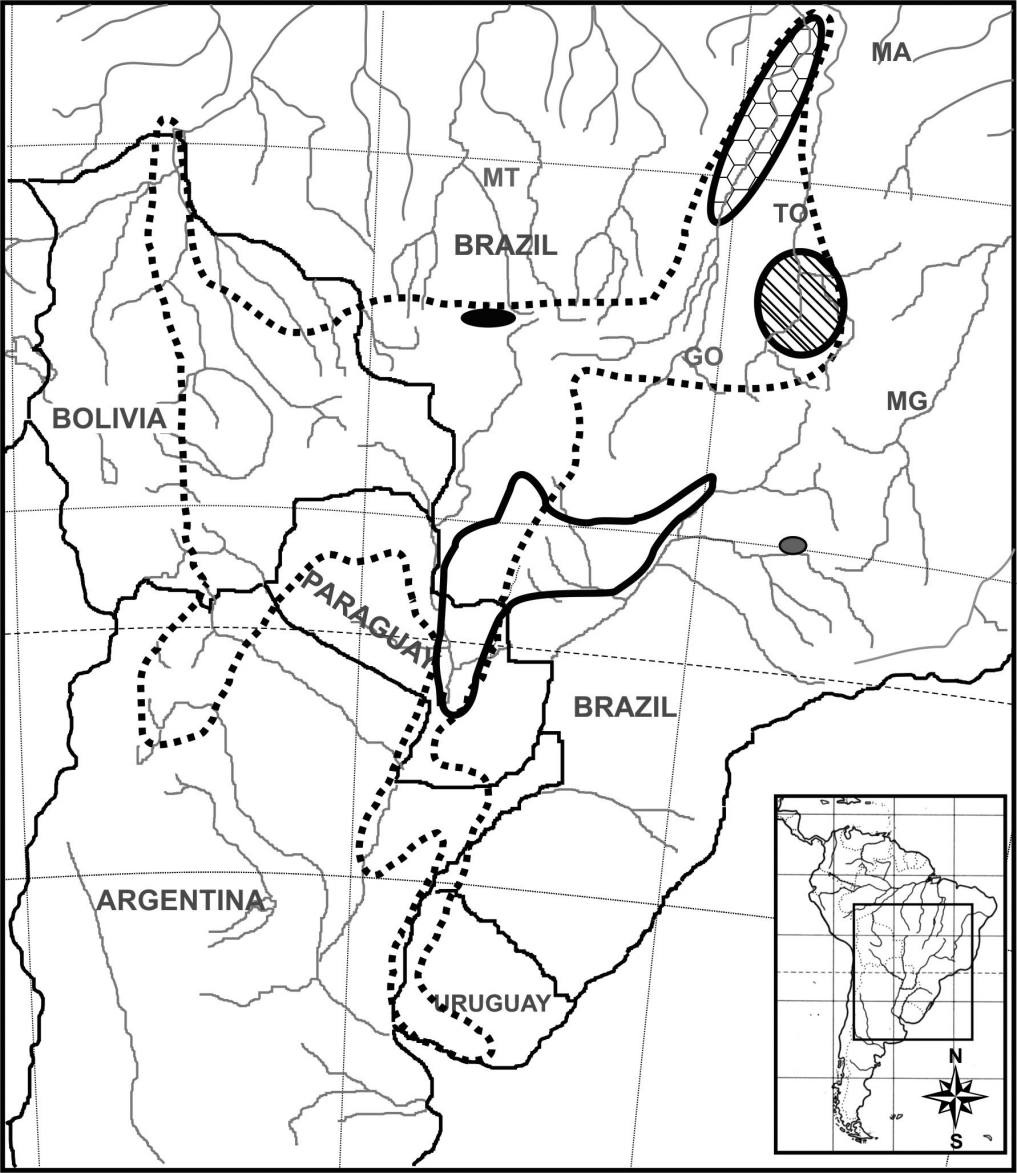
Geographic distribution of *Arachis* species with x=9. *Arachis decora* – diagonal pattern; *Arachis palustris* – octagon pattern; *Arachis praecox* – black field; *Arachis porphyrocalyx* – grey field. Dashed gray line indicates the distribution of section Arachis and solid gray line the distribution of Erectoides section.

The only known population of *Arachis porphyrocalyx* is located in the state of Minas Gerais (Brazil), near to the Rio Grande, 20 km southeast of Uberaba. Taking into account the geographic areas of the sections described by Krapovickas and Gregory (1994), this location is outside the geographic area of the section Erectoides (Fig. 1).

*Arachis porphyrocalyx* has thickened secondary roots, flowers mostly at the base of the lateral branches, and presents anthocyanin in the flower calyx, characteristics for which it has been included in the section Erectoides (Valls and Simpson 2005). Also, this species has a perennial life cycle. However, the authors clarify that the above-ground growth of this species resembles that of *Arachis villosa* (Benth, 1841) of the section Arachis (Valls and Simpson 2005). Several molecular analyses have been done to understand the genetic relationships between *Arachis* species but only one includes *Arachis porphyrocalyx* (Hoshino et al. 2006). This analysis of microsatellite markers placed this species within the cluster of species of section Erectoides but forming a subcluster together with *Arachis vallsii* (Krapov. & W.C. Gregory, 1994) of the section Arachis (Lavia 2001, Lavia et al. 2009), *Arachis subcoriacea* (Krapov. & W.C. Gregory, 1994) of the section Procumbentes and *Arachis dardani* (Krapov. & W.C. Gregory, 1994) of the section Heteranthae (Hoshino et al. 2006). Therefore, the taxonomic position of *Arachis porphyrocalyx* is not well established.

Moreover, the chromosome data on this species are very peculiar. Peñaloza and Valls (2005) noted that the karyotype of *Arachis porphyrocalyx* includes subtelocentric chromosomes, which is uncommon in the genus, and it has satellite chromosomes (SAT chromosomes) type 8 based on the appreciation of the metaphases. Futhermore, Lavia (2008) noted that a pair of chromosomes of this species behaves like the ‘A’ chromosomes, which is a peculiarity of the species with the A genome of section Arachis (Fernández and Krapovickas 1994, Lavia 1996, Robledo et al. 2009). Consequently, the possible presence of this chromosome pair in *Arachis porphyrocalyx* would be a quite relevant difference from x = 9 species of the section Arachis and raises the question about the relationships of this species with those of section Arachis.

In this context, in the present work, we analysed the presence of ‘A’ chromosomes using classical cytogenetics on mitotic prometaphases and metaphases, the distribution patterns of C-DAPI+ heterochromatin in the karyotype and the mapping of the ribosomal gene loci by FISH to (i) confirm the presence of ‘A’ chromosomes in *Arachis porphyrocalyx*, (ii) build a detailed cytogenetic map, (iii) investigate their karyotype relationships with the x = 9 species of the section Arachis by analysing chromosome homologies and finally (iv) discuss if the origin of all *Arachis* species with x = 9 is single or multiple. The chromosome data provided in this analysis will improve the knowledge of the genome affinities between the wild species, therefore aiding in understanding the variability contained in the secondary gene pool of the most agronomically important species of genus: *Arachis hypogaea* (Linneaus, 1753) (peanut).

## Material and methods

### Plant material

The material studied of *Arachis porphyrocalyx* corresponds to accession J.F.M. Valls, J.P. Moss and G.P. Silva 7303, collected in Brazil, Minas Gerais state, municipality of Uberaba, in the gardens of the Uberaba Country Club, on the edge of highway BR-050, next to Río Grande river, 20 km southeast of Uberaba, 19°58'S 47°47'W, in 1983. Germplasm from this original collection has been conserved at the Wild Arachis Genebank of Embrapa, in Brasília, Distrito Federal, and increased seed has been distributed to partner institutions. Seeds used in this study were obtained from the peanut germplasm collections of the Instituto de Botánica del Nordeste in Corrientes, Argentina. The voucher materials of the original accession are deposited in the herbaria CTES and CEN, and are paratypes of the species name. The holotype and isotypes of *Arachis porphyrocalyx* were collected nine years later from exactly the same site (J.F.M. Valls, C.E. Simpson, R.N. Pittman, D.E. Williams and G.P. Silva 13271).

### Chromosome preparations and staining

#### Feulgen staining

Roots were obtained from seeds germinated in pots under laboratory conditions. Healthy root apices (5–10 mm long) were pretreated with 2 mM 8-hydroxyquinoline for 3 h at room temperature (Fernández and Krapovickas 1994). Subsequently, they were fixed in 3:1 absolute ethanol:glacial acetic acid for 12 h at 4°C and stored at -20°C until use. For conventional chromosome staining, fixed root apices were washed in distilled water for 5 min, hydrolysed in 1 N HCl for 8 min at 60°C, stained with Schiff’s reagent (Feulgen’s technique) and then squashed in a drop of 2% acetic orcein. The preparations were made permanent using Euparal as mounting medium.

#### rDNA detection and DAPI banding

Fixed root apices were digested in 1% (w/v) cellulose (from *Trichoderma viridae*; Onozuka R-10, Serva) plus 10% (v/v) pectinase (from *Aspergillus niger*, Sigma) dissolved in 40% glycerol in 0.01 M citrate buffer (pH 4.8) for 2 h at 37°C. Subsequently, the meristematic cells were removed from the root tip and squashed in 45% acetic acid. After remove of the coverslip with gas carbon dioxide, the slides were air dried, aged for 1–2 days at room temperature and then kept at -20°C until use.

#### Probe labelling and fluorescence in situ hybridization

The 5S and 45S rDNA loci were localised using probes pA5S, pA18S and pA26S isolated from genomic DNA of *Arachis hypogaea* (Robledo and Seijo 2008) and labelled by nick translation with digoxigenin-11-dUTP (Roche Diagnostics) or biotin-11-dUTP (Sigma-Aldrich). Pretreatment of slides, chromosome and probe denaturation, conditions for the in situ hybridisation (hybridisation mixes contained DNA probes at a concentration of 2.5–3.5 ng/µl, with a stringency to allow sequences with 80%–85% identity to remain hybridized), post-hybridization washing, blocking and indirect detection with fluorochrome-conjugated antibodies were performed according to Seijo et al. (2004). The first set of antibodies consisted of anti-biotin produced in goat (Sigma-Aldrich) and monoclonal anti-digoxigenin conjugated to fluorescein isothiocyanate (FITC) produced in mouse (Sigma-Aldrich). The second set consisted of anti-goat conjugated to tetramethyl-rodamine isothiocyanate (TRITC) produced in rabbit (Sigma-Aldrich) and anti-mouse conjugated to FITC produced in sheep (Sigma-Aldrich). Preparations were counterstained by mounting them with Vectashield medium (Vector Laboratories) containing 2 mg/ml of 4',6-diamidino-2-phenylindole (DAPI).

#### Fluorescent microscopy and image acquisition

Chromosomes were viewed with a Leica DMRX fluorescence microscope (Leica) and digitally photographed with a computer-assisted Leica DC 350 digital camera system. Red, green and blue images were captured in black and white using the respective filters for TRITC, FITC and DAPI excitations. Digital images were processed with PHOTOSHOP, version 7.0 (Adobe).

### Karyotype analysis

Karyotype measures were obtained by the analysis of five individuals and four Feulgen-stained metaphase plates per individual and using the computer application MICROMEASURE version 3.3 (Reeves and Tear 2000). Karyotype description is based on the nomenclature by Levan et al. (1964). Chromosomes were classified in three categories according to the centromeric index (CI = short arm × 100/total length of chromosome): metacentric (m) when CI = 50–37.5 and submetacentric (sm) when CI = 37.5–25 and subtelocentric when CI = 25–12.5. Classification of SAT chromosomes was performed on the basis of the satellite relative size and position of the centromere (Fernández and Krapovickas 1994). The total chromosome length (TCL) was obtained by summing the average length of each chromosome in the four metaphase samples of each individual, and then the average of the five individuals was performed. The chromosome mean length was calculated by dividing the TCL by the number of chromosomes of the species. The karyotype asymmetry indices were estimated using the intrachromosomal (A_1_) and interchromosomal (A_2_) indexes by Romero Zarco (1986).

Data from homologous chromosomes were combined first to obtain mean values of different pairs of chromosomes in the same metaphases and, subsequently, of the same chromosome pair in different metaphases. Haploid complements were represented in the ideogram. Chromosomes were ordered first by morphology and then by decreasing size.

## Results and discussion

**Figure 2.**
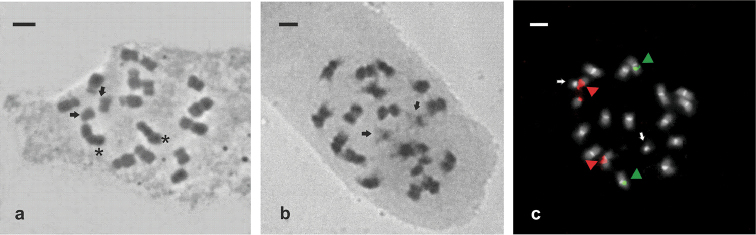
Mitotic chromosomes of *Arachis porphyrocalyx*. **a–b** Feulgen technique **c** double fluorescent *in situ* hybridization (FISH). **a** Metaphase displaying 2n=18, the starts indicate satellites and the arrows indicate the pair of “A chromosomes” **b** Prometaphase showing the pair of “A” chromosomes indicated by arrows **c** The yellow-green and red signals correspond to the 5S and 45S rDNA loci, and the white correspond to the heterochromatin bands C-DAPI+ after FISH. The arrows indicate the pair of “A chromosomes”. Bar = 3 µm.

**Figure 3.**
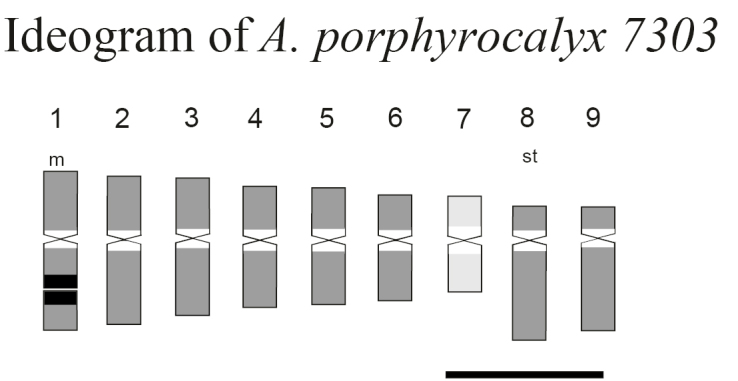
Ideogram of *Arachis porphyrocalyx* performed with measures of chromosomes obtained by classical technique. The A chromosome is represented with light gray colour. Distribution of 5S rDNA loci is illustrated with a striped signal and 18S-26S rDNA loci with a black signal. Heterochromatic regions counterstained with C-DAPI+ are represented with white bands. Bar = 2 µm.

General karyotype features, karyotype formula, presence of ‘A’ chromosomes, TCL, mean chromosome length, centromeric index, asymmetry indexes, number of chromosomes with heterochromatic DAPI+ bands and number and position of 5S and 45S rDNA loci for *Arachis porphyrocalyx* are listed in Table 1. To compare with the remaining species with *x* = 9 from the section Arachis, the chromosome data of *Arachis decora*, *Arachis palustris* and *Arachis praecox* (Lavia 1998, Lavia 1999, Silvestri et al. 2015) were included in this table. Representative somatic prometaphases and metaphases of *Arachis porphyrocalyx* are shown in Figure 2, and the respective ideogram is shown in Figure 3.

**Table 1.** Karyotypical features in x = 9 species of the genus *Arachis*.

Species	Karyotype formula	A chromosomes	Total chromosome length (µm)	Chromosome length mean (µm)	CI	Asymmetry indexes		Number of chromosomes with DAPI+ bands	Number and position of rDNA loci	
						A_1_	A_2_		45S	5S
*Arachis porphyrocalyx*	14m + 4st	yes	29.37	1.63	41.60	0.30	0.16	18	one LA pair 1	one LA pair 2
*Arachis decora*	16m + 2sm^b^	no	33.66^b^	1.87^b^	45.41^b^	0.22^b^	0.16^b^	18^c^	one LA pair 9^c^	one SA pair 6^c^
*Arachis palustris*	16m + 2sm^a^	no	33.23^a^	1.85^a^	43.64^a^	0.22^a^	0.17^a^	16^c^	one LA pair 9^c^	one SA pair 6^c^
*Arachis praecox*	16m + 2sm^a^	no	35.28^a^	1.96^a^	43.47 ^a^	0.23^a^	0.12^a^	18^c^	one LA pair 9^c^	one SA pair 6^c^

Abbreviations: CI = centromeric index ; A1 = Intrachromosomal asymmetry index ; A2 = interchromosomal asymmetry index . m = metacentric , sm = submetacentric , st = subtelocentric , LA = long arm , SA = short arm . Data of *Arachis decora*, *Arachis palustris* and *Arachis praecox* were taken from Lavia (1998)^a^, Lavia (1999)^b^ and Silvestri et al. (2015)^c^.

### General characteristics of karyotypes

The chromosome number of *Arachis porphyrocalyx*, previously determined by Peñaloza and Valls (2005), was confirmed by the mitotic analysis, 2*n* = 2*x* = 18 (Fig. 2a). Likewise, in all analysed metaphases, a chromosome pair with characteristics of ‘A’ chromosomes was observed (Fig. 2b). This chromosome pair was easily identified by showing a lower condensation level of the euchromatin regions of their arms in comparison with the same regions in the remaining chromosomes of the complement and by corresponding to the smallest chromosomes of the karyotype. The ‘A’ chromosome pair corresponds to pair 7 (Fig. 3).

The karyotype consisted of seven pairs of metacentric chromosomes and two subtelocentric pairs (14m + 4st; Fig. 3). These data do not agree with those reported by Peñaloza and Valls (2005) who observed four pairs of submetacentric chromosomes, but this discrepancy can be due to the fact that the formula published by these authors is based only on a visual analysis. Chromosomal size ranged between 1.24 and 2.08 µm with a mean length of 1.63 µm, belonging to the category of small chromosomes according to Lima de Faría (1980), and the mean length of diploid complement was 29.37 µm (Table 1). The indexes of asymmetry revealed a moderately high degree of intrachromosomal asymmetry (A1 = 0.30) but low interchromosomal asymmetry (A2 = 0.16). Only one pair of SAT chromosomes was found in all analysed metaphases. These chromosomes were the longest metacentric chromosomes of the complement (pair 1) and, as previously reported by Peñaloza and Valls (2005), correspond to the SAT chromosomes type 8 described by Fernández and Krapovickas (1994).

The metaphases of some individuals showed two or three chromosome pairs with extended primary constrictions (centromeres) and the chromosome arms separated. These chromosomes had the centromere unusually large or stretched during prophase or prometaphase, and consequently the number of chromosomal elements increased up to 25. Similar behaviour has been observed in some chromosomes of other species of the genus *Arachis*, such as *Arachis cardenasii* (Krapov. & W.C. Gregory, 1994), *Arachis helodes* (Mart. ex Krapov. & Rigoni, 1958), *Arachis valida* (Krapov. & W.C. Gregory, 1994), *Arachis duranensis* (Krapov. & W.C. Gregory, 1994) and *Arachis correntina* ((Burkart) Krapov. & W.C. Gregory, 1994) all belonging to the section Arachis (Fernández and Krapovickas 1994), and of other genera such as *Antirrhinum majus* (Linneaus, 1753), *Allium sphaerocephalum* (Crome ex Schltdl, 1824) (Lima De Faria 1956), *Libocedrus chilensis* (Endlicher, 1847) (Hunziker 1961), *Pisum* (Linneaus, 1753) and *Lathyrus* (Linnaeus, 1753) (Neumann et al. 2015). Just recently, Neumann et al. (2015) have classified this type of centromere as ‘intermediate’ between the two types of centromeres, monocentric and holocentric, and having an organization characterized by multiple Cen-H3 domains.

DAPI staining after FISH revealed C-DAPI+ centromeric bands in all chromosomes of the karyotype (Fig. 2c). These bands had similar sizes along karyotype, except in the ‘A’ chromosomes, where they were most conspicuous (Fig. 3). The results of in situ hybridisation showed one pair of 45S rDNA loci in proximal position on the long arm of the longest metacentric chromosomes (pair 1) and one pair of 5S rDNA loci in proximal or interstitial positions on the second longest chromosome pair of the karyotype (pair 2; Figs 2c and 3).

### Chromosome homeologies of *Arachis porphyrocalyx* with the remaining species of the genus, particularly with x = 9 species.

Like most species of the genus *Arachis*, the karyotype of *Arachis porphyrocalyx* consists of small size chromosomes, mainly metacentric. The smallest chromosome pair showed all features that define the ‘A’ chromosomes: a chromosome length 54% smaller than the largest chromosomes of karyotype, and showing allocycly in somatic prophases and pro-metaphases (Fernández and Krapovickas 1994). Chromosomes with these features have not been reported until now for other species not assigned to the A genome of the section Arachis (Fernández and Krapovickas 1994, Robledo et al. 2009). *Arachis porphyrocalyx* has a moderately asymmetric karyotype due to the presence of two pairs of subtelocentric chromosomes. This structure contrasts with the more symmetric karyotypes of the other *x* = 9 species of *Arachis* that are composed by eight metacentric pairs and only one submetacentric (Lavia 1998, 1999). Besides, the presence of subtelocentric chromosomes distinguishes *Arachis porphyrocalyx* from the other species of the section Erectoides, which have karyotypes formed only by metacentric and submetacentric chromosomes (Fernández and Krapovickas 1994, Lavia 2001, Lavia et al. 2009, Ortiz et al. 2013). Indeed, the presence of subtelocentric chromosomes in the karyotypes is uncommon within the genus *Arachis*, a feature that *Arachis porphyrocalyx* only shares with *Arachis batizocoi* (Krapov. & W.C. Gregory, 1974) and *Arachis glandulifera* (Stalker, 1991) (Fernández and Krapovickas 1994), both species of the section Arachis.

Even though *Arachis porphyrocalyx* owns a unique pair of SAT chromosomes in metaphase, as the other species with *x* = 9, these chromosomes correspond to a different type according to the classification proposed by Fernández and Krapovickas (1994). Thus, *Arachis porphyrocalyx* has SAT chromosomes type 8, while the remaining *x* = 9 species show type 3 (Lavia 1998, Silvestri et al. 2015). Similarly, it differs from those observed in *Arachis douradiana* (Krapov. & W.C. Gregory, 1994), *Arachis hermannii* (Krapov. & W.C. Gregory, 1994), *Arachis major* (Krapov. & W.C. Gregory, 1994), *Arachis paraguariensis* (Chodat & Hassler, 1904) and *Arachis stenophylla* (Krapov. & W.C. Gregory, 1994) from the section Erectoides that have satellites of type 2, 3A or 4 (Fernández and Krapovickas 1994, Lavia 2001, Lavia et al. 2009, Ortiz et al. 2013).

Until now, four distribution patterns of centromeric C-DAPI+ heterochromatin have been identified in the karyotypes of *Arachis* species (Seijo et al. 2004, Robledo and Seijo 2008, 2010, Robledo et al. 2009, Silvestri et al. 2015, Ortiz, unpublished). One of them, proper to *Arachis glandulifera* of section Arachis (D genome), is characterised by a markedly heterogeneous distribution, with chromosomes showing large bands, and others with faint bands or devoid of them. The second pattern, with large bands of similar size in all or almost all chromosome pairs, is present in the *x* = 9 species and A and K genome species of the section Arachis. The third pattern, with small blocks at most chromosomes, which are revealed as faint bands or like-dot bands on the flanks of the centromeres, is shown in the F genome species of the section Arachis and in species of sections Erectoides and Procumbentes. And the fourth possibility, with no detectable bands in the entire karyotype, is proper to B genome species of the section Arachis. *Arachis porphyrocalyx* has a pattern that is different from those species of the section Erectoides and is similar to that observed in the *x* = 9 and in some *x* = 10 species of the section Arachis. It even resembles that observed in the A genome species, since the A chromosomes have pericentromeric bands with relative size greater than those in the rest of karyotype; which also in turn strengthens the identity of these chromosomes.

Regarding the number and location of ribosomal loci (45S rDNA and 5S rDNA), *Arachis porphyrocalyx* has the same number of sites as other *x* = 9 species, that is one pair of each loci (Silvestri et al. 2015). However, the 45S rDNA loci in *Arachis porphyrocalyx* are located on a metacentric pair as in *Arachis praecox*, while in *Arachis palustris* and *Arachis decora*, they are located on a submetacentric pair. Meanwhile, 5S rDNA loci in *Arachis porphyrocalyx* are on the long arms of a large-size metacentric pair and in the remaining *x* = 9 species on the short arms of a small-size metacentric pair. Until now, the number of rDNA loci has been characterized for two species of the section Erectoides, *Arachis stenophylla* and *Arachis paraguariensis* (Raina and Mukai 1999). The number of ribosomal loci observed in *Arachis porphyrocalyx* agrees with what has been detected in those species, except that *Arachis stenophylla* has two pairs of 45S rDNA loci.

The fact that the karyotype of *Arachis porphyrocalyx* has distinct distribution pattern of heterochromatin, conformed by large bands of the similar size in all chromosome pairs, and has SAT chromosomes type 8 suggests that it corresponds to a distinct genome from that present in Erectoides species. On the contrary, its banding pattern is most related to that present in *x* = 9 species of the section Arachis. However, due to the presence of a pair of A chromosomes, different SAT chromosomes, different location of the 5S rDNA loci and a more asymmetric karyotype than that of the other *x* = 9 species, it is suggested that *Arachis porphyrocalyx* also does not have the G genome.

### Has the basic chromosome number x = 9 in *Arachis* been originated once or more times in the evolutionary history of the genus?

Although the four *x* = 9 species share the chromosome number, the karyotypic differences between *Arachis porphyrocalyx* and the remaining three species are evident. Therefore, and as was proposed (Peñaloza and Valls 2005), the reduction in the number of chromosomes might have occurred more than once in the *Arachis* genus.

All *Arachis* species with *x* = 9 are naturally distributed in Brazil (Fig. 1), and their evolutionary history probably is associated with watercourses. The northernward distribution corresponds to *Arachis palustris* and comprises both sides of Tocantins River in the states of Maranhão and Tocantins, between 7°22'S and 12°33'S. *Arachis decora* is distributed in the northeast of Goiás and in the south of Tocantins state, separated by approximately 150 km from *Arachis palustris*. In contrast, the only two populations known to *Arachis praecox* are located in the Mato Grosso state, separated by approximately 900 km of any of the other two *x* = 9 species of the section Arachis. Meanwhile, *Arachis porphyrocalyx* has been located in Minas Gerais state, municipality of Uberaba, near the Rio Grande, some 20 km southeast of Uberaba (19°58'S, 47°47'W). Thus, the four *x* = 9 species of the genus *Arachis*, at least in the present, belong to three different basins (Fig. 1). That is, *Arachis decora* and *Arachis palustris* share the Tocantins River Basin and are the closest species from the geographic standpoint; *Arachis praecox* belongs to the basin of the Paraguay River, while *Arachis porphyrocalyx* to the Paraná River. Notoriously the last species is located in the same basin that lodges the species with A chromosomes of section Arachis (Robledo et al. 2009).

The three *x* = 9 species of section Arachis are annuals and morphologically constitute different entities. *Arachis praecox* differs from *Arachis palustris* and *Arachis decora* by the short central axis from 2 to 3 cm, while in the other two it has about 15 cm of length. *Arachis decora* and *Arachis palustris* are morphologically very similar although they are distinguished because the former has bristles in the stipules, while the latter lacks them (Krapovickas and Gregory 1994). Fruit shape also distinguishes these two species. Contrarily, *Arachis porphyrocalyx* is perennial, has thickened secondary roots, a central axis between 5 and 15 cm of length, flowers concentrated at the base of the plant, lateral branches procumbents, epiphyllum with hairs, petioles and rachis with hairs and bristles (Valls and Simpson 2005), a series of features that lead to its initial allocation in the Erectoides section.

As previously discussed, *Arachis porphyrocalyx* does not share the same genome of the other *x* = 9 species. The presence of A chromosomes would be a strong reason for the assignment of *Arachis porphyrocalyx* to A genome, but the fact that it has two subtelocentric chromosomes, SAT chromosomes type 8, a single pair of 45S ADNr sites, and as the most significant trait, the basic number *x* = 9 distinguishes it from the three karyotype types established for A genome species (Robledo et al. 2009).

Taking into account the geographic distribution, morphological and cytogenetic features, the hypothesis of Peñaloza and Valls (2005), which suggests the basic chromosome number *x* = 9 would have originated at least twice in the evolutionary history of the genus, becomes relevant.

Some years ago, when the existence of a diploid *x* = 9 species with a pair of A chromosomes was not yet known, it had been proposed that a diploid *x* = 10 species, belonging either to the A genome group (Lavia 1998) or to a non-A genome group (Tallury et al. 2005), was the ancestor of all species with *x* = 9 by reduction of chromosome number. In this work, it has been demonstrated that *Arachis porphyrocalyx* has not the same genome type of the other *x* = 9 species, suggesting that the reduction of the number of chromosomes must have occurred more than once in the evolution of the genus *Arachis*; therefore, the proposed hypotheses must be updated.

**Figure 4.**
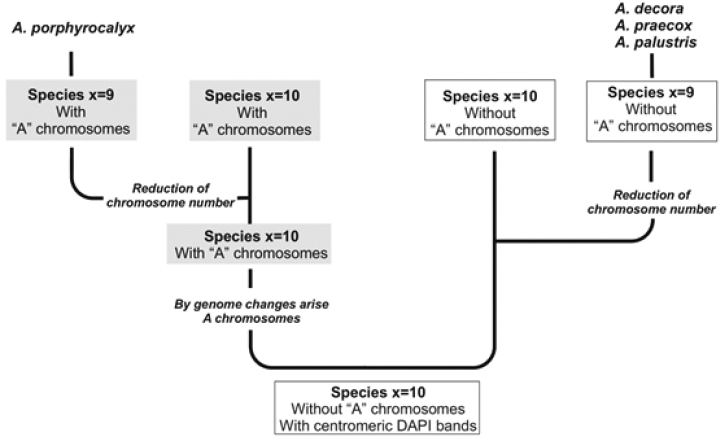
Scheme showing the hypothesis of the multiple origin of x = 9 species with and without A chromosomes in the genus *Arachis*.

In this sense, we propose that a diploid x = 10 entity, without A chromosomes and with large bands of the similar size in all or almost all, chromosome pairs could be the common ancestor of all x = 9 species as well as the x = 10 species with A and K genome of the section Arachis. The fact that these species share a same type of heterochromatin distribution pattern, different from that observed in the species so far examined of the genus Arachis, would support this proposal. From this ancestor, by chromosomal rearrangements, an entity with A chromosomes has been originated, from which all x = 10 species with A chromosomes (A genome species) derived, and by some cytogenetic phenomenon (probably disploidy), the unique species with x = 9 and A chromosomes (*Arachis porphyrocalyx*) derived (Fig. 4). Moreover, new molecular data (Silvestri; unpublished) show a minor genetic distance of *Arachis porphyrocalyx* from the A genome species, compared with any other species of the genus, including the rest of x = 9 species. In parallel, from that same first common ancestor, an evolutionary line of species without A chromosomes continued evolution, and from this, the x = 9 species with the G genome is derived by reducing the number of chromosomes (Fig. 4). Evidence in this direction results in several phylogenetic analyses (Bechara et al. 2010, Friend et al. 2010, Moretzshon et al. 2013), in which the x = 9 species appear as a sister clade to the species without A chromosomes of the section Arachis.

## Conclusions

In this work, we confirmed the presence of ‘A’ chromosomes in the karyotype of *Arachis porphyrocalyx*. It revealed its particular karyotypic structure, which allows proposing that it does not share the same genome with the remaining *x* = 9 species of *Arachis* and neither with the species so far characterized karyotypically of the section Erectoides. On the contrary, its similarity with karyotypes of species with A chromosomes of the section Arachis suggests that the genome of *Arachis porphyrocalyx* could be related to the A genome, but molecular studies are needed to confirm this hypothesis. Additionally, considering the morphological and cytogenetic features and the geographic distribution, we propose the existence of two separate events for the origin of species with 18 chromosomes within the genus *Arachis*.
