# Corrigenda: Genomic characterisation of *Arachis porphyrocalyx* (Valls & C.E. Simpson, 2005) (Leguminosae): multiple origin of *Arachis* species with x = 9. Comparative Cytogenetics 11(1): 29–43. doi: 10.3897/CompCytogen.v11i1.10339

**DOI:** 10.3897/CompCytogen.v11i4.21560

**Published:** 2017-12-08

**Authors:** Silvestri María Celeste, Alejandra Marcela Ortiz, Germán Ariel Robledo, José Francisco Montenegro Valls, Graciela Inés Lavia

**Affiliations:** 1 Instituto de Botánica del Nordeste (CONICET-UNNE, Fac. Cs. Agrarias), Sargento Cabral 2131, C.C. 209, 3400 Corrientes, Argentina; 2 Facultad de Ciencias Exactas y Naturales y Agrimensura, UNNE, Av. Libertad 5460, 3400 Corrientes, Argentina; 3 Embrapa Recursos Genéticos e Biotecnologia, Brasília, DF, Brasil

After the publication of our article, we detected an error in the figure 3. On the ideogram, the signal of 5S rDNA loci illustrated with a striped signal over the long arm of chromosome 2 at proximal/interstitial position is not observed. Correct figure is as follows:

**Figure 3.**
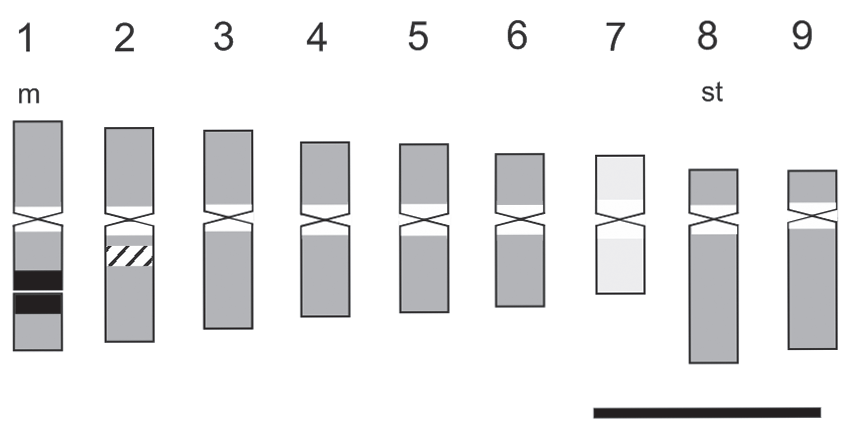
Ideogram of *A. porphyrocalyx* performed with measures of chromosomes obtained by classical technique. The “A” chromosome is represented with light grey colour. Distribution of 5S rDNA loci is illustrated with a striped signal and that of 18S–26S rDNA loci with a black signal. Heterochromatic regions counterstained with C-DAPI+ are represented with white bands. Scale = 2 μm.

